# Data Reconstruction Using Smart Sensor Placement

**DOI:** 10.3390/s24186008

**Published:** 2024-09-17

**Authors:** Farnaz Boudaghi, Danial Waleed, Luis A. Duffaut Espinosa

**Affiliations:** Department of Electrical and Biomedical Engineering, University of Vermont, Burlington, VT 05405, USA; fboudagh@uvm.edu (F.B.); dwaleed@uvm.edu (D.W.)

**Keywords:** sensor placement, QR placement method, optimal number of sensors, model-free control, Kriging Kalman Filter

## Abstract

This paper deals with spatio-temporal field estimation with efficient sensor placement based on the QR decomposition. The proposed method also identifies the optimal number of sensors needed for field estimation that captures the most relevant features of the field of interest. To address the uncertainties inherent in spatio-temporal field estimation, a robust data-driven control method is utilized, providing resilience against unpredictable environmental and model changes. In particular, the approach uses the Kriged Kalman Filter (KKF) for uncertainty-aware field reconstruction. Unlike other reconstruction methods, the positional uncertainty originating from the data acquisition platform is integrated into the KKF estimator. Numerical results are presented to show the efficacy of the proposed dynamic sensor placement strategy together with the KKF field estimator.

## 1. Introduction

Recent advancements in sensor technology have paved the way for a wide range of applications and services in areas encompassing safety and surveillance, climate prediction, and the environmental monitoring of pollution and temperature. These sensors are distributed across various locations and work together as a network. The primary task of these networks is to perform distributed data sampling, essentially sensing the environmental conditions to gather valuable information. Collecting data is an even more expensive and inefficient endeavor when such collection needs to be performed periodically. The challenge thus lies in optimizing sensor placement and movement to ensure accurate data collection with minimal resources. This involves using fewer sensors to cover large areas efficiently and relying on smart algorithms to determine the optimal positions. Placing sensors strategically allows for the creation of sensing patterns over space and time, ensuring that specific performance criteria such as energy efficiency, data quality, the accuracy of estimates, and the likelihood of detection are met [1,2,3,4,5,6,7].

Complex controlled systems benefit from a systematic approach to selecting the most convenient outputs so that their states can be reconstructed/estimated reliably. For a system constituting a sensing network, these outputs can be defined in terms of the positioning of the network members in places that provide a better representation of spatial information of the phenomenon at hand. Some other applications of state reconstruction using sensor data include controlling fluid flows [8], optimizing power grids [9], and enhancing high-performance computing [10]. Data-driven system approaches such as those based on Koopman theory also rely on sensor placement methods to enhance estimation and control [11,12]. In this context, the Kalman Filter (KF) framework allows us to assimilate the information over time to enhance the reconstruction of high-dimensional states using a minimal number of sensors [13,14]. Among the plethora of sensor placement methodologies, QR sensor placement has been applied across a range of measurement selection scenarios due to its efficiency and the fact that it maximizes the conditioning of reconstructing transformations [15,16,17,18,19]. Thus, this technique is integral to the contribution of this paper.

A dynamic sensor network cannot disregard the dynamics of the platforms carrying the sensor of interest. This is even more important when such dynamics can change during monitoring missions in harsh environments. In this context, Model-Free Control (MFC) is an innovative non-linear control technique that adopts a data-driven approach to construct a linear surrogate model that replaces the complex dynamics of controlled systems, which allows control laws to change on the fly when the system unexpectedly changes [20,21,22,23,24]. This surrogate model, commonly known as the *ultra-local model* (ULM), encompasses two key parameters. The first parameter captures the overall time-varying dynamics of the system. The second parameter is considered as a tuning knob and is determined through expert knowledge of the system, thus employing a heuristic approach that is generally based on the system’s characteristics. Additionally, the system error dynamics is highly simplified, such that a control law can be designed for the purpose of stability and improving performance. The theoretical foundation of MFC was established in [20], aiming to eliminate the requirement for an accurate model and precise knowledge of system parameters for effective control. In particular, the time-varying parameter in the ULM adaptively captures the overall system’s dynamics during a short time interval. Thus, this parameter is updated at every time step to make sure that the unmodeled dynamics of the operated system can be captured effectively. MFC demonstrates its versatility through a wide array of applications. For instance, MFC was applied to a gantry crane system whose outputs are unreliable but can recover performance under a Kalman Filter robustification approach [21]. Furthermore, MFC has been successfully applied to control a quadcopter system but without the uncertainty quantification of the ULM performance or its position [22,25]. This uncertainty quantification is part of the work presented in this paper.

Various methodologies can be employed in the implementation of MFC when estimating the ULM. Traditionally, digital filters have been a common choice for this purpose [20]. Another approach involves the use of a Luenberger observer, where the designer can tailor the convergence speed of the ultra-local model by adjusting the selected gains [26]. Despite their success, these methodologies share a limitation: they do not provide an uncertainty quantification of the parameters or state of the system. Such enhancement can be carried out by incorporating a KF approach for estimating the ULM, as in [21,27]. This approach offers the advantage of quantifying the positional uncertainty generated from the MFC methodology in the form of the corresponding covariance matrix needed for the subsequent integration with spatial reconstruction methodologies.

Monitoring the spatial and temporal changes in a field with a minimal number of sensors mounted on systems controlled using MFC while achieving a specific performance constitutes a problem within the field of sensor networks [28]. Environmental, geophysical, and biological processes, which are known for their complex variability in space and time, can be modeled based on their spatial and temporal correlations for stationary fields. For non-stationary fields, dynamic models help describe their evolution [29]. When a field exhibits both stationary and non-stationary traits, a Kriged Kalman Filter (KKF) [30], constituted by the merging of Kriging [29,31] with a KF, is known to effectively monitor these changes. Specifically, the KKF has been shown to be able to reconstruct spatial fields of information [32]. Combining a minimum sensor-placement problem with a KKF can efficiently improve the reconstruction of a particular information field by decreasing the required points from which data need to be obtained. This becomes particularly advantageous in challenging environments like the Arctic, where data acquisition presents its own set of hurdles. The use of unmanned aerial vehicles (UAVs) gathering data from these critical points offers a viable solution to the problem. Moreover, MFC proves valuable in this context due to its ability to adapt to both external environmental and internal dynamic changes.

Methods such as those in [2,29,31,32] have been employed for the reconstruction of spatial fields, but these techniques do not take into account the positional uncertainty of the locations where data are taken. Additionally, the KKF does not provide an optimal procedure for deciding which locations for data acquisition are best given a dynamic field of information. While there is work on finding optimal sensor locations such as in [15,16,17,18,19], their integration with the KKF is lacking. Lastly, MFC has been implemented for various applications [20,21,27,33,34,35,36]; however, there is no application to our knowledge using the uncertainty obtained from MFC in spatial reconstruction methodologies. Therefore, the main contributions of this paper are as follows.

i.

We provide an algorithm that determines the optimal number of points essential for reconstructing a given field. This procedure will be referred to as the optimal sensor placement procedure.

ii.

We integrate MFC on quadcopters to collect data from selected locations. MFC aims to enhance system robustness in the face of the challenging external conditions that may be encountered during terrain reconstruction in harsh environments.

iii.

We implement MFC employing a Kalman Filter (MFC-KF) to estimate the ULM for quadcopters. This method quantifies the position uncertainty of the sensing platform.

iv.

We augment the covariance obtained from the MFC-KF with that of the KKF to improve the reconstruction process. This integration takes into account the variance associated with the sensors used for data acquisition, further refining the accuracy of the reconstructed field.

The rest of the paper is organized as follows. Section 2 provides the preliminaries of the techniques used in the proposed method (QR sensor placement, MFC, and a description of the KKF). Section 3 presents the proposed methodology that integrates the concepts described in the preliminary section. Section 4, provides numerical simulations that illustrate the proposed methodology with synthetic information. The final section gives the conclusions.

## 2. Preliminaries

In this section, we will first focus on compressed sensing in order to provide a foundation for how data reconstruction can be achieved by selecting key locations for measurements. This will be followed by sensor placement, which will focus on how to find the key data points in a set in order to reconstruct the data. Then, a brief summary of MFC will be provided that mainly focuses on how MFC based on the Kalman Filter (MFC-KF) works. This is followed by details on a robustification technique that can be used to make systems using MFC resilient to contaminated measurements. The robustification processes provides details on how to obtain reliable data and, more importantly, the quantification of the positional uncertainty. In this paper, the reconstruction of the data is carried out by quadcopters; therefore, a background on the modeling of a quadcopter is presented. Lastly, details on the KKF method are provided.

### 2.1. Compressed Sensing

The compressibility of most natural signals, like images and audio, implies that, in a suitable basis, only a limited number of modes are active. Consequently, compressed sensing leverages a signal’s sparsity on a universal basis to accomplish complete signal reconstruction from remarkably few measurements. Similarly, many high-dimensional physical systems can be effectively described by a low-dimensional attractor, enabling efficient prediction and control. States evolving through nonlinear dynamics often have compact representations in an appropriate transform basis. A compressible signal (i.e., a state) $x\in R^{n}$ can be represented as

(1) x=Ψϱ,

where ϱ is a sparse vector indicating the few active modes of $\Psi \in R^{n\times n}$ [18,37]. More specifically, the vector ϱ is referred to as *K*-sparse within the basis Ψ when it contains exactly *K* non-zero elements. In scenarios where the basis Ψ is generic, such as the Fourier or wavelet bases, only a few active elements in ϱ are essential for reconstructing the original signal *x*. This sparsity allows for an efficient low-rank representation of the transform basis, $\Psi_{r}\in R^{n\times r}$, for the sparse low-rank coefficients $a\in R^{r}$, which can be written as

(2) $$x=\Psi_{r}a.$$

By collecting significantly fewer selected measurements, it becomes feasible to solve for the non-zero elements of ϱ within the transformed coordinate system. One can then define the measurements $y_{s}\in R^{p}$, where p≪n, as 

(3) $$y_{s}=C_{s}x,$$

where $C_{s}\in R^{p\times n}$ selects *p* measurements of the signal *x*. The selection of the measurement matrix $C_{s}$ holds paramount significance in compressed sensing, which is one of the challenges in this study. Moreover, within compressed sensing, the objective is to identify the sparsest vector ϱ that aligns with the provided measurements $y_{s}$. Combining (1) and (3), it follows that

(4) $$y_{s}=\left(C_{s}\Psi \right)\varrho =\Theta \varrho .$$

The selection of $C_{s}$ aims to ensure that the operator Θ is well conditioned for reconstructing *x* out of $y_{s}$. In the case that some expert knowledge of the signal is known, custom sensors can be crafted specifically for those signals using the Proper Orthogonal Decomposition (POD). The POD identifies key features from data and represents high-dimensional signals using a limited set of orthonormal eigenmodes, forming a lower-dimensional embedding space, and allows efficient data handling. Thus, for instance, Singular Value Decomposition (SVD) can be used for reconstructing the complete signal from the low-dimensional representation while optimizing accuracy and minimizing errors.

### 2.2. Sensor Placement and the QR Factorization

The goal of sensor placement is to choose sensor positions that capture a low- dimensional subspace with significant characteristics to reconstruct high-dimensional signals efficiently. Such positions, $\gamma =[\gamma_{1}\;\gamma_{2}\;\cdots \;\gamma_{p}]$, are encoded in $C_{s}$, as mentioned before. In the often scenario where the vector *x* is unknown, the basis coefficients *a* in (2) are approximated as

(5) $$\hat{a}=\Theta^{-1}y_{s}={\left(C_{s}\Psi_{r}\right)}^{-1}y_{s}.$$

Note that (5) is only valid when the number of measurements is equal to the number of significant modes in the basis $\Psi_{r}$ (p=r), for p>r, and we have $\hat{a}={\left(\Theta^{T}\Theta \right)}^{-1}\Theta^{T}y_{s}$.

The most effective reconstruction of *x* is achieved by pinpointing the rows of $\Psi_{r}$ that correspond to optimal sensor locations. Since Θ might not be square and depends on the choice of γ, we denote $M_{\gamma }:=\Theta$ when p=r and $M_{\gamma }:=\Theta^{T}\Theta$ when p≠r. The problem can thus be formulated by finding γ such that the minimum singular value of $M_{\gamma }$, $\sigma_{se_{min}}$, is maximized. That is, we need to solve

(6) $$\gamma^{*}=\underset{\gamma ,|\gamma |=p}{arg min}{∥M_{\gamma }^{-1}∥}_{2}=\underset{\gamma ,|\gamma |=p}{arg max}\sigma_{se_{min}}\left(M_{\gamma }\right).$$

The QR factorization is a fundamental numerical technique in linear algebra that decomposes a matrix into an orthogonal matrix (Q) and an upper triangular matrix (R) [38]. Equation (6) is solved using QR factorization. QR factorization with column pivoting introduces a permutation matrix where the pivoting locations define γ and, therefore, gives a $C_{s}$ that enhances the numerical stability and accuracy of the reconstruction process. Thus, the QR factorization with column pivoting selects *p* locations that most effectively sample the *r* basis modes of $\Psi_{r}$.

### 2.3. Model-Free Control

Since the objective is to reconstruct information with data acquired from a sensing platform operating in uncertain environments, the dynamics of the systems carrying the sensors must be accounted for. Consider an arbitrary system with *n* dimensions, governed by the following differential equation:

(7) $$H_{m}\left(y_{m},{\dot{y}}_{m},...,y_{m}^{\left(n_{y}\right)},u,\dot{u},...,u^{\left(n_{u}\right)}\right)=0,$$

where $H_{m}$ is a function that encapsulates the relationships among the system’s inputs, outputs, and their respective derivatives ($n_{y}$ derivatives of $y_{m}$ and $n_{u}$ derivatives of *u*). A significant challenge arises in formulating $H_{m}$ due to the inherent lack of complete knowledge about the system. This limitation often originates from unmodeled dynamics, uncertainties in modeled parameters, and unforeseen changes in the model dynamics due to operating in harsh environments.

To address these challenges, one effective approach is to employ a surrogate model. This model estimates the overall time-varying dynamics of the system using input–output data obtained from sensors in the system. One such surrogate model is the ULM. The ULM expresses the system dynamics for a short period of time as

(8) $$y_{m}^{\left(v\right)}=F+\beta u,$$

where *v* is the order of the ULM, *F* represents the overall time-varying dynamics that need to be estimated, β is a tuning parameter, and *u* is the input going into the system [20,21,26]. Note that there does not exist a systematic methodology for selecting β, and expert knowledge of the application is often required to select or tune such a value. Nevertheless, some attempts have been proposed [27,39], where β is estimated using data-driven approaches. This work assumes that the selection of β is performed using expert knowledge. Therefore, the idea of MFC is to introduce a control law *u* that cancels the time-varying dynamics and add an additional internal controller to produce a compensating control effort $u_{c}$ for reducing tracking error. That is,

(9) $$u=-\frac{F-y_{d}^{\left(v\right)}+u_{c}}{\beta },$$

where $y_{d}^{\left(v\right)}$ is the *v*-th derivative of the desired reference trajectory. Traditionally, the control effort is implemented via a simple PID (Proportional, Integral, and Derivative) control strategy or one of its variants, PI, PD, and P. Due to the nature of MFC and its ability to adapt to time-varying dynamics, this implementation is often referred to as intelligent iPID, iPI, iPD, and iP [20]. Substituting (9) into (8) leads to

(10) $$y_{m}^{\left(v\right)}=F+\beta \left(-\frac{F-y_{d}^{\left(v\right)}+u_{c}}{\beta }\right).$$

Simplifying (10) gives

(11) $$y_{m}^{\left(v\right)}=y_{d}^{\left(v\right)}-u_{c}.$$

**Figure 1:**
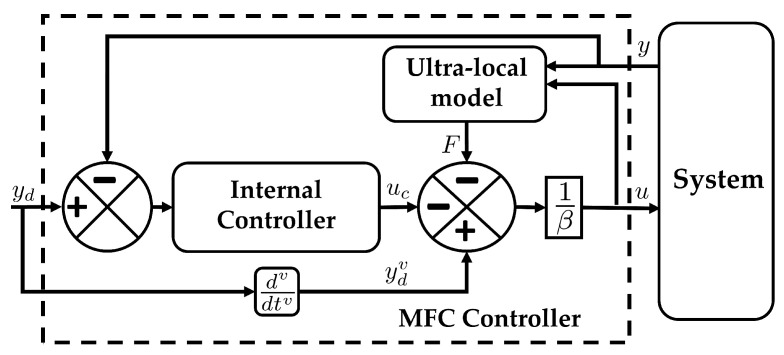
Implementation of MFC for a system.

Defining $e^{\left(v\right)}=y_{d}^{\left(v\right)}-y_{m}^{\left(v\right)}$, (11) can be written as $e^{\left(v\right)}=u_{c}$, where the control effort $u_{c}$ can be easily designed to minimize $e^{\left(v\right)}$. The choice of *v* is based on the type of controller used. For example, if an iPID or iPD is used, then v=2. On the other hand, if iPI is required, then v=1. A basic implementation of MFC on a generic system is given in Figure 1.

Traditionally, the estimation of the ULM parameters is performed using digital filter [20,22,40]. Other methodologies include the use of Luenberger observers [26], where the ULM given in (8) is given the form of a linear state space representation. On the other hand, a KF approach can also be used to estimate the parameters of the ULM as shown in [21]. First, (8) is converted into a state space system. Assuming v=2, (8) in continuous time is expressed as

(12) $${\dot{z}}_{m1}=z_{m2},\;\;{\dot{z}}_{m2}=F+\beta u.$$

A first-order discretization of (12) gives

(13) $$\begin{array}{cc} z_{m1[k+1]} & =z_{m1[k]}+T_{s}z_{m2[k]}+\frac{1}{2}T_{s}^{2}\left(F_{k}+\beta u_{k}\right),\\ z_{m2[k+1]} & =z_{m2[k]}+T_{s}\left(F+\beta u_{k}\right). \end{array}$$

To estimate *F* at time *k*, a new state $z_{m3}$ is introduced, and (13) is augmented as

(14) $$\begin{array}{cc} z_{m1[k+1]} & =z_{m1[k]}+T_{s}z_{m2[k]}+\frac{1}{2}T_{s}^{2}\left(z_{m3[k]}+\beta u_{k}\right),\\ z_{m2[k+1]} & =z_{m2[k]}+T_{s}\left(z_{m3[k]}+\beta u_{k}\right),\\ z_{m3[k+1]} & =z_{m3[k]}, \end{array}$$

which assumes that $z_{m3}$ does not change too fast. The steps for the KF using the ULM are then:

*i*. Prediction step:
  (15a) $${\hat{z}}_{m[k|k-1]}=A_{d}{\hat{z}}_{k-1}+B_{d}u_{k},$$
  (15b) $$P_{k|k-1}^{-}=A_{d}P_{k-1|k-1}^{+}A_{d}^{T}+Q_{k}.$$
  where
  $$A_{d}=\;\left[\begin{array}{ccc} 1 & T_{s} & \frac{T_{s}^{2}}{2}\\ 0 & 1 & T_{s}\\ 0 & 0 & 1 \end{array}\right],B_{d}=\;\left[\begin{array}{c} \frac{T_{s}^{2}\beta }{2}\\ T_{s}\beta \\ 0 \end{array}\right],C_{d}=\;\left[\begin{array}{ccc} 1 & 0 & 0\\ 0 & 1 & 0 \end{array}\right],\;\mathrm{and}\;{\hat{z}}_{m[k]}=\;\left[\begin{array}{c} z_{m1[k]}\\ z_{m2[k]}\\ z_{m3[k]} \end{array}\right]\;\;.$$
ii. Correction step:
  (16a) $$K=P_{k|k-1}^{-}C_{d}{\left(C_{d}P_{k|k-1}^{-}C_{d}^{T}+R_{k}\right)}^{-1},$$
  (16b) $${\hat{y}}_{k}=C_{d}{\hat{z}}_{m[k|k-1]},$$
  (16c) $${\hat{z}}_{m[k|k]}={\hat{z}}_{m[k|k-1]}+K\left(y_{k}-{\hat{y}}_{k}\right),$$
  (16d) $$P_{k|k}^{+}=P_{k|k-1}^{-}-KC_{d}P_{k|k-1}^{-},$$

where $Q_{k}=E\left\{w_{k}w_{k}^{T}\right\}$, $R_{k}=E\left\{v_{k}v_{k}^{T}\right\}$, $v_{k}$ represents the standard deviation of the sensor noise,  $w_{k}$ is the standard deviation of the process noise, *K* is the Kalman gain, $P^{-}$ is the a priori covariance, $P^{+}$ is the a posteriori covariance, and $y_{k}$ is the output obtained from the system [21].

### 2.4. Robustification of MFC

Since MFC is a data-driven approach, it is prone to measurement corruption or sensor failure. This is even more of a problem when MFC is used in harsh or unknown environments. Therefore, a robustification of MFC can be performed by integrating the *Robust Generalized Maximum-Likelihood Kalman Filter* (RGMKF) [21,41,42] into the MFC estimation methodology of the ULM. One of the key advantages of using RGMKF is its capability to handle process, sensor, and structural outliers while estimating a system’s states [41]. In other words, the RGMKF offers a procedure for the rectification of states and their corresponding covariances by reducing the impact of outliers on the estimation process. The RGMKF operates through three main steps; the first consists of formulating the redundant observation vector. Here, the data coming from the system are stacked together. That is,

(17) $${\tilde{z}}_{k}={\left[y_{k}\;\;{\hat{z}}_{k|k-1}^{-}\right]}^{T},$$

where $y_{k}$ are the sensor measurements coming from the system and ${\hat{z}}_{k|k-1}^{-}$ are state predictions coming from (15a). The vector $y_{k}$ consists of multiple sensor values for each state and the observation matrix for $\tilde{z}$ is $\tilde{C}={\left[C_{r}\;\;I\right]}^{T}$, where $C_{r}$ has as many rows as the number of redundant sensors. The second step consists of detecting outliers in (17), which is performed via Projection Statistics (PS) [43]. Before obtaining the best estimate of the system state from (17), one needs to pre-white the information to uncorrelate the data from the noise in the system. The data are pre-whitened as

(18) $${\tilde{y}}_{k}^{pr}=G_{k}{\tilde{z}}_{k},$$

where $G_{k}$ is obtained via Cholesky decomposition of the noise covariance matrix associated with (17). The next step estimates the system states via the Iterative Reweighted Least Squares method. That is,

(19) $${\tilde{x}}_{k|k}^{\left(n_{i}+1\right)}={\left({\tilde{C}}_{k}^{⊤}Q^{\left(n_{i}\right)}{\tilde{C}}_{k}\right)}^{-1}\;\;\;{\tilde{C}}_{k}^{⊤}Q^{\left(n_{i}\right)}{\tilde{y}}_{k}^{pr},$$

where the recursion is performed over the weight matrix $Q^{\left(n_{i}\right)}$. More detail on the RGMKF can be found in [21,41,42]. Once the states have been rectified, the last step consists of correcting (16d). That is,

(20) $${\tilde{P}}_{k|k}^{+}=1.03{\left({\tilde{C}}_{k}^{⊤}{\tilde{C}}_{k}\right)}^{-1}\left({\tilde{C}}_{k}^{⊤}Q_{w}{\tilde{C}}_{k}\right){\left({\tilde{C}}_{k}^{⊤}{\tilde{C}}_{k}\right)}^{-1}$$

where $Q_{w}$ is a diagonal matrix containing the PS weights is used on the quadcopters comprising the sensor network. As previously mentioned, robustification through the RGMKF is achieved by incorporating additional sensors, which can increase operational costs. A potential solution, as demonstrated in [42], is to introduce redundancy through a swarm-based approach. However, this type of redundancy is not pursued here since it is outside the scope of the paper.

In the next subsection, a brief background on the modeling of a quadcopter system is provided.

### 2.5. Quadcopter System

**Figure 2:**
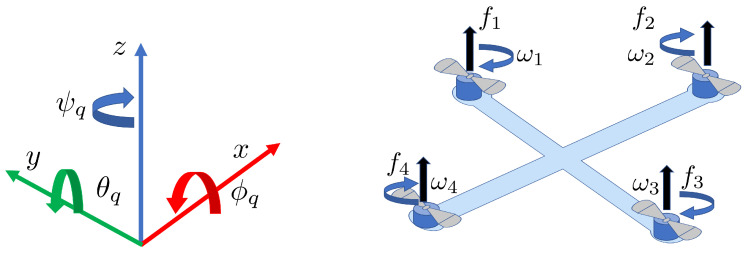
Quadcopter model showing all six degrees of freedom.

The dynamic of a quadcopter is usually obtained based on force/moment dynamics and kinematics, with additional insights available in [22,25,44]. That is,  

(21) $$\begin{array}{c} \left[\begin{array}{c} {\ddot{\phi }}_{q}\\ {\ddot{\theta }}_{q}\\ {\ddot{\psi }}_{q}\\ {\ddot{x}}_{q}\\ {\ddot{y}}_{q}\\ {\ddot{z}}_{q} \end{array}\right]=\left[\begin{array}{c} \left(-k_{dp}{\dot{\phi }}_{q}^{2}+\left(I_{yy}-I_{zz}\right){\dot{\theta }}_{q}{\dot{\psi }}_{q}-J\omega_{r}{\dot{\theta }}_{q}+u_{2}\right)/I_{xx}\\ \left(-k_{dq}{\dot{\theta }}_{q}^{2}+\left(I_{zz}-I_{xx}\right){\dot{\phi }}_{q}{\dot{\psi }}_{q}+J\omega_{r}{\dot{\phi }}_{q}+u_{3}\right)/I_{yy}\\ \left(-k_{dr}{\dot{\psi }}_{q}^{2}+\left(I_{xx}-I_{yy}\right){\dot{\phi }}_{q}{\dot{\theta }}_{q}+u_{4}\right)/I_{zz}\\ \left(-k_{dx}{\dot{x}}_{q}+\left(c\left(\psi_{q}\right)s\left(\theta_{q}\right)c\left(\phi_{q}\right)+s\left(\psi_{q}\right)s\left(\phi_{q}\right)\right)u_{1}\right)/m\\ \left(-k_{dy}{\dot{y}}_{q}+\left(s\left(\psi_{q}\right)s\left(\theta_{q}\right)c\left(\phi_{q}\right)-c\left(\psi_{q}\right)s\left(\phi_{q}\right)\right)u_{1}\right)/m\\ \left(-k_{dz}{\dot{z}}_{q}-mg+\left(c\left(\theta_{q}\right)c\left(\phi_{q}\right)\right)u_{1}\right)/m \end{array}\right], \end{array}$$

where s=sin, c=cos, a (x,y,zdn) represent the position of the quadcopter in the Earth frame and $\left(\phi_{q},\theta_{q},\psi_{q}\right)$ are the rotation angles in the body frame. Additionally, $k_{d}\left[x,y,z\right]$ represents the translation drag along each axis, $k_{d}\left[p,q,r\right]$ are the rotational drag coefficients, $\omega_{r}$ is the difference between the rotor angular velocities, and *J* is the rotor’s moment of inertia. A simple schematic of (21) is shown in Figure 2, where the lift force generated by each motor is denoted by $f_{i}$, and the angular velocity generated by each motor is denoted by $\omega_{i}$ with i∈{1,2,3,4}. The inputs to the system in terms of forces and angular velocities are

$$\begin{array}{c} u_{1}=\sum_{i=1}^{4}f_{i}=K_{m}\cdot \sum_{i=1}^{4}\omega_{i}^{2}\\ u_{2}=l\left(f_{4}-f_{2}\right)=K_{m}\cdot l\left(\omega_{4}^{2}-\omega_{2}^{2}\right)\\ u_{3}=l\left(f_{1}-f_{3}\right)=K_{m}\cdot l\left(\omega_{1}^{2}-\omega_{3}^{2}\right)\\ u_{4}=d\left(f_{2}+f_{4}-f_{1}-f_{3}\right)=K_{m}\cdot d\left(\omega_{2}^{2}+\omega_{4}^{2}-\omega_{1}^{2}-\omega_{3}^{2}\right)\\ \omega_{r}=\omega_{1}-\omega_{2}+\omega_{3}-\omega_{4}, \end{array}$$

where $K_{m}$ is a force velocity constant, *l* is the length of the arm, and *d* is the rotor’s reaction torque constant. When the model of the quadcopter is available, the traditional approach is to convert (21) into control affine form [45]. However, since MFC is being used, there is no need for this control-affine form in the formulation of appropriate quadcopter control laws. It is, however, important to emphasize that (21) is presented here to show the intricacies of the acquisition network dynamics and that the model is solely employed for obtaining simulation results in subsequent sections while the inputs used to drive the quadcopter to the desired locations are provided by the robustified MFC-KF method. In the next section, a brief introduction to the KKF algorithm is provided since a modified KKF technique will subsequently be developed for reconstructing a field of information with the awareness of positional uncertainty originating from the acquisition network dynamics.

### 2.6. Kriged Kalman Filter

The Kriged Kalman Filter (KKF) is a technique designed for reconstructing spatially and temporally varying fields. This method integrates two well-established techniques: Kriging and the KF. Kriging is used for estimating unobserved data based on observed values from nearby locations, leveraging the spatial correlation among data points [30,32]. The KF estimates the states of a system by considering the statistical noise, observed values, and a model of the system [46]. By combining these approaches, the KKF effectively manages the complexities of spatiotemporal data, providing accurate and robust estimates. The steps of KF have been covered in Equations (15) and (16), and similar steps are used in this section showing how to integrate the two techniques.

Consider field dynamics (e.g., produced by meteorological quantities such as wind or terrain variability such as snow with respect to historical bare land conditions). Thus, the spatially distributed information of interest at time *k* is assumed to satisfy a dynamical equation of the form

(22) $$\begin{array}{c} {\hat{y}}_{k}=H_{k}\;{\hat{y}}_{k-1}^{+}+w_{k}, \end{array}$$

where ${\hat{y}}_{k}$ denotes the predicted spatial information of the field parametrized by position $x_{k}$ (i.e., ${\hat{y}}_{k}={\hat{y}}_{k}\left(x_{k}\right)$ and ${\hat{y}}_{k-1}={\hat{y}}_{k-1}\left(x_{k}\right)$), ${\hat{y}}_{k}^{+}$ is the prior best estimate of the field for time k−1, $H_{k}$ is the redistribution of parameters from time k−1 to time *k*, and $w_{k}$ is the Gaussian process noise associated with the spatial information with the covariance matrix $\Sigma_{Q}=E\left\{w_{k}w_{k}^{T}\right\}$. The translation and diffusion of the spatial transition dynamics $H_{k}$ can be modeled as

(23) $${\left(H_{k}\right)}_{ij}=\epsilon_{k}\;e^{-{\left(x_{ik}-x_{jk}a_{k}^{ij}\right)}^{⊤}{\left(D_{k}^{ij}\right)}^{-1}\left(x_{ik}-x_{jk}a_{k}^{ij}\right)}$$

where $\epsilon_{k}\in \left(0,1\right)$ is a parameter aiming to keep (22) stable and $a_{k}^{ij}\in R$ and $D_{k}^{ij}\in R^{+}$ are the translation and dilation parameters of an appropriate Gaussian kernel, respectively. The latter is related to a simple discretization of an advection and diffusion process [1]. The sparse prediction of the measurements can be introduced in a formulation via the observation equation

(24) $$z_{k}=C_{k}{\hat{y}}_{k}+\eta_{k},$$

where $C_{k}\in {\left\{0,1\right\}}^{N_{z}^{2}\times N^{2}}$, $\eta_{k}$ is Gaussian noise associated with the measured values, and $\Sigma_{E}=E\left\{\eta_{k}\eta_{k}^{T}\right\}$ is the associated covariance. Observe that the size of the matrix $C_{k}$ varies with time, where $N_{z}$ is the number of measurements taken over the sparse field. The matrix $C_{k}$ is obtained by assuming the identity $C_{k}^{⊤}C_{k}=\mathrm{diag}\left(\kappa_{k}\right)$, where $\kappa_{k}\in {\left\{0,1\right\}}^{N_{z}}$ with 1 denoting the locations in $y_{k}$ where measurements were taken and 0 otherwise. Notice that $C_{k}$ is the same matrix used for sensor placement in Section 2.1 but time-varying. Let $\Sigma_{\iota [k]}^{-}$ be the a priori covariance for the field dynamics prediction at time *k*. Its propagation follows as

(25) $$\Sigma_{\iota [k]}^{-}=H_{k}\Sigma_{\iota [k-1]}^{+}H_{k}^{⊤}+\Sigma_{Q}.$$

The measurements covariance, $R_{k}$, is given by

(26) $$\begin{array}{c} R_{k}=C_{k}\Sigma_{Q}C_{k}^{⊤}+\Sigma_{E[k]} \end{array}$$

with $\Sigma_{E[k]}=\sigma_{e}I_{N_{z}}$ being the assumed noise caused by the process of sparsely measuring the field of information. The KKF Kalman gain can now be given as

(27) $$L_{k}=\Sigma_{\iota [k]}^{-}C_{k}^{⊤}{\left(C_{k}\Sigma_{\iota [k]}^{-}C_{k}^{⊤}+R_{k}\right)}^{-1},$$

and the field correction by

(28) $${\bar{y}}_{k}={\hat{y}}_{k}+L_{k}\left(z_{m,k}-C_{k}\mu_{k}-z_{k}\right)$$

where $z_{m,k}$ values are the measurements obtained (which can come from an arbitrary system capable of selecting data points within a field system such as a quadcopter), $\mu_{k}$ is the spatial trend of the information field (for instance, obtained from ancillary information or historical data), and $z_{k}$ is the prediction from information at time k−1 in (22). The covariance correction step follows naturally as

(29) $$\Sigma_{\iota [k]}^{+}=\left(I_{N}-L_{k}C_{K}\right)\Sigma_{\iota [k-1]}^{-}.$$

Next, the Kriging step for the stationary part of the field is  

(30) $${\bar{y}}_{s,k}=\mu_{k}+\Sigma_{Q}C_{k}^{⊤}R_{k}^{-1}\left(z_{m,k}-C_{k}{\bar{y}}_{k}-C_{k}\mu_{k}\right).$$

The final KKF step involves putting together the field update with the Kriging step of the stationary information. That is,

(31) $$y_{k}^{*}={\bar{y}}_{k}+{\bar{y}}_{s,k}.$$

## 3. Methodology

This section focuses on providing a method for making the KKF uncertainty-aware. First, an optimal sensor placement algorithm that identifies the minimal number of sensors required to reconstruct a field accurately is given. Next, the robustified MFC-KF for a quadcopter system is presented. Finally, the methodology for extracting the positional uncertainty of the quadcopter system and its integration with the KKF is provided.

### 3.1. Optimal Number of Sensors

**Figure 3:**
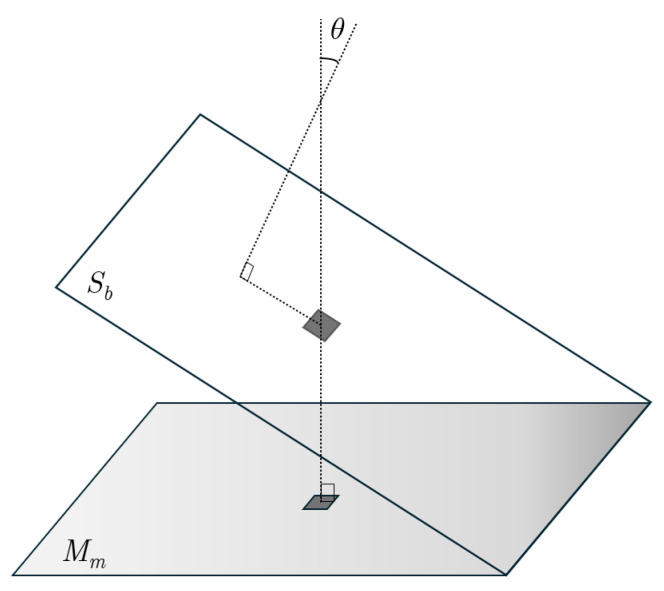
Relative positions of the low-dimensional subspace $S_{b}$ and the space of measurements $M_{m}$.

As mentioned earlier, managing distributed processing systems effectively requires strategies for estimating unseen states due to the high cost and limitations of accessible sensors. Efficiency is achieved by identifying a minimal set of measurements that approximate the entire field of interest through a low-dimensional system representation. The goal is to capture significant characteristics within the measurement space, influenced by the eigenvalues of the matrix Θ in (4). Figure 3 shows a geometric visualization of the estimation problem. In this depiction, θ represents the angle between the measurement subspace $M_{m}$ and the low-dimensional space $S_{b}$. Small angles indicate that estimates are accurate modulo the intrinsic error produced by the dimension reduction. On the contrary, wider angles can substantially diminish the precision of the estimation to the extent of becoming unreliable. The nonzero singular values, $\sigma_{se_{i}}$ of Θ can be interpreted in terms of the angles formed by vectors in the subspaces $S_{b}$ and $M_{m}$, with the relationship given by $\cos\theta =\sigma_{se}$ [47].

Here, the focus is on reducing the dimensionality of a measurement data set by determining the optimal number of measurements and their location required to capture the most relevant features of the field. Let $M_{\gamma ,p}$ be the result of the QR sensor placement for a choice of *p* sensors, and let $\sigma_{se_{p}}$ be the corresponding maximized lowest singular value. Then, finding $p^{*}\in \left\{1,\ldots ,m\right\}$, where *m* is the total number of possible measurements, such that $\sigma_{se_{p^{*}}}>\sigma_{se_{p}}$ for all $p\neq p^{*}$, provides the subspace with the smallest angle with respect to the measurement space. Therefore, we need to solve  

(32) $$p^{*}=\underset{p\in \{1,\ldots ,m\}}{arg max}\sigma_{se_{p}}\left(M_{\gamma }\right).$$

Observe that (32) is specific to the QR placement methodology since $\sigma_{se_{p}}$ is obtained directly from $M_{\gamma^{*},p}$ and obviously depends on $\gamma^{*}$, which defines $C_{s}$. A simple algorithm solving (32) is given in Algorithm 1.   
**Algorithm 1:** Algorithm for Optimal Sensor Placement with Optimal Number of Sensors (OSPN)
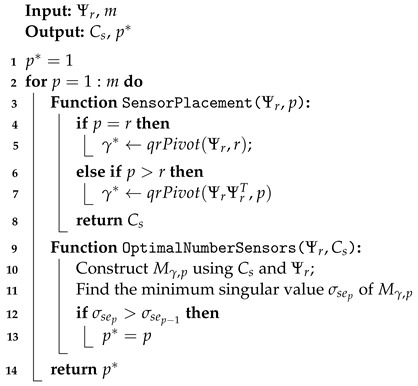


### 3.2. Implementation of MFC on a Quadcopter System

**Figure 4:**
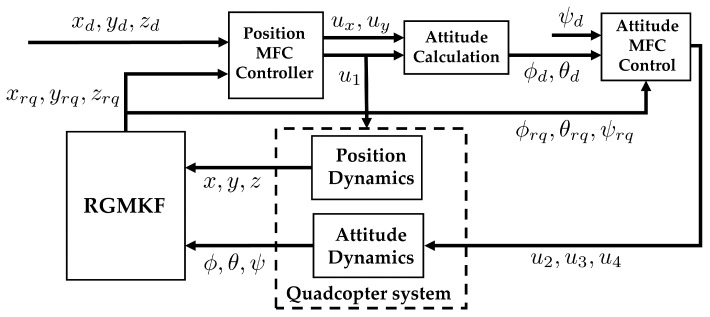
Overall integration of MFC with a quadcopter system using cascade control scheme.

Since quadcopters are underactuated systems, a common approach to controlling quadcopters is to use a cascade control scheme [25,44]. In this methodology, the desired trajectory is used to calculate the desired roll $\phi_{d}$ and pitch $\theta_{d}$. Integrating an MFC with quadcopter systems follows a similar strategy. A block diagram for such integration is given in Figure 4. Here, the desired $x_{d},\;y_{d}$, and $z_{d}$ are fed to the position controller, and the position control generates $u_{x},\;u_{y}$ and $u_{1c}$. These three control efforts, along with the current yaw $\psi_{q}$ of the quadcopter, are fed to the attitude calculator, which generates the desired roll $\phi_{d}$ and pitch $\theta_{d}$. Next, the desired attitude is then fed to the attitude controller to calculate the desired control effort $u_{2c},u_{3c}$, and $u_{4c}$. The combined control efforts are then fed to the quadcopter system. The sensor values coming from the quadcopter system are then fed to the RGMKF for rectification. The integration of MFC with RGMKF is referred to as MFC-RGMKF. The new rectified states are then fed to the respective controller where the subscript rq is used to highlight that the states have been rectified.

The position controller and the attitude controller are based on the MFC method, which follow the control scheme shown in Figure 1. MFC here uses v=2, and the internal controller is a simple PD controller. The overall control effort being generated by the scheme is

(33) $$\begin{array}{c} u_{x}=\frac{1}{\beta_{x}}\left(-F_{x}+{\ddot{x}}_{d}-Kp_{x}\left(e_{x}\right)-Kd_{x}\left({\dot{e}}_{x}\right)\right),\\ u_{y}=\frac{1}{\beta_{y}}\left(-F_{y}+{\ddot{y}}_{d}-Kp_{y}\left(e_{y}\right)-Kd_{y}\left({\dot{e}}_{y}\right)\right),\\ u_{1}=\frac{1}{\beta_{z}}\left(-F_{z}+{\ddot{z}}_{d}-Kp_{z}\left(e_{z}\right)-Kd_{z}\left({\dot{e}}_{z}\right)\right),\\ u_{2}=\frac{1}{\beta_{\phi }}\left(-F_{\phi }+{\ddot{\phi }}_{d}-Kp_{\phi }\left(e_{\phi }\right)-Kd_{\phi }\left({\dot{e}}_{\phi }\right)\right),\\ u_{3}=\frac{1}{\beta_{\theta }}\left(-F_{\theta }+{\ddot{\theta }}_{d}-Kp_{\theta }\left(e_{\theta }\right)-Kd_{\theta }\left({\dot{e}}_{\theta }\right)\right),\\ u_{4}=\frac{1}{\beta_{\psi }}\left(-F_{\psi }+{\ddot{\psi }}_{d}-Kp_{\psi }\left(e_{\psi }\right)-Kd_{\psi }\left({\dot{e}}_{\psi }\right)\right). \end{array}$$

Note that $u_{x}$ and $u_{y}$ are internal inputs produced by the MFC-RGMKF and that $u_{i}$ for i=1,…4 are the inputs to the quadcopter, as shown in Figure 4. Therefore, using the position dynamics of ${\ddot{x}}_{q}$ and ${\ddot{y}}_{q}$ described in (21), the only MFC equations relating $\phi_{d}$ and $\theta_{d}$ to the system inputs are

(34) $$\left[\begin{array}{c} {\ddot{x}}_{q}\\ {\ddot{y}}_{q} \end{array}\right]=\left[\begin{array}{c} F_{x}\\ F_{y} \end{array}\right]+\left[\begin{array}{c} \beta_{x}\\ \beta_{y} \end{array}\right]R_{\psi }\left[\begin{array}{c} \sin\phi_{q}\\ \sin\phi_{q}\cos\theta_{q} \end{array}\right]u_{1},$$

where $R_{\psi }=\left[\begin{array}{cc} \sin\psi_{q} & \cos\psi_{q}\\ -\cos\psi_{q} & \sin\psi_{q} \end{array}\right]$ and $u_{1}$ is the same as in (21). The last step in the control scheme is the connection between the MFC position control and the MFC attitude control. This is shown as the attitude calculation block in Figure 4, whose objective is to give $\phi_{d}$ and $\psi_{d}$. For this purpose, let the control effort generated by the position control for the *x* and *y* positions be redefined as follows:

$$\left[\begin{array}{c} {\ddot{x}}_{q}\\ {\ddot{y}}_{q} \end{array}\right]=\left[\begin{array}{c} u_{x}\\ u_{y} \end{array}\right]=R_{\psi }\left[\begin{array}{c} \sin\phi_{d}\\ \sin\phi_{d}\cos\theta_{d} \end{array}\right]u_{1}.$$

Then, via simple inversion, it follows that

$$\left[\begin{array}{c} \sin\phi_{d}\\ \sin\phi_{d}\cos\theta_{d} \end{array}\right]=\frac{1}{u_{1}}R_{\psi }^{-1}\left[\begin{array}{c} u_{x}\\ u_{y} \end{array}\right]=:\left[\begin{array}{c} {\bar{u}}_{x}\\ {\bar{u}}_{y} \end{array}\right],$$

which allows one to obtain the desired $\phi_{d}$ and $\theta_{d}$ as

(35) $$\begin{array}{cc} \; & \phi_{d}=\arcsin\left({\bar{u}}_{x}\right)\;\mathrm{and}\;\theta_{d}=\arcsin\left(\frac{{\bar{u}}_{y}}{\cos(\phi_{d})}\right). \end{array}$$

### 3.3. Quantification of Positional Error via MFC-RGMKF

To improve the field reconstruction given by the KKF, the uncertainty inherently present in the acquisition sensor network needs to be considered. The KKF reconstruction, as described in Section 2.6, assumes that measurements taken within the region of interest are accurate without any errors and that the sole source of uncertainty stems from the reconstruction processes. Therefore, one needs to integrate these two sources of uncertainty into the reconstruction processes. This process involves extracting covariance data from the drone at specified positions and integrating these covariances into the KKF for error quantification.

The positions of interest are determined using the sensor placement methodology outlined in Section 3.1. The quadcopters comprising the acquisition network navigate to these locations, utilizing the inputs given by the MFC-RGMKF. Note that MFC-RGMKF not only produces the inputs of the quadcopters without a precise model but also provides a measure of uncertainty at each time step through the quantification of the positional and velocity covariances. Algorithm 2 outlines the procedure to obtain the covariances accounting for the mentioned uncertainty quantification [48].
**Algorithm 2:** Quadcopter Planning and Covariance Quantification
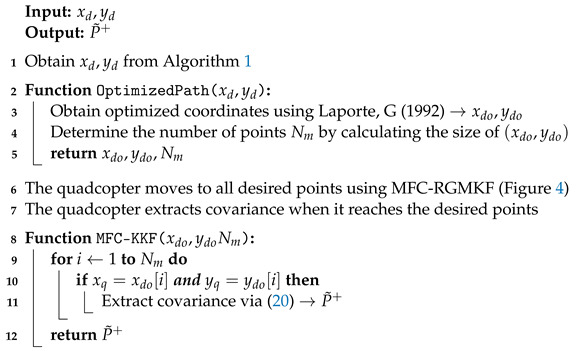


To integrate the covariance obtained from the drone with KKF, first assume that the location of field observations of the acquisition network is given by a random variable $X_{k}$ and that a measurement outcome of $X_{k}$ is $\xi_{k}$. Thus, the covariance of $X_{k}$ given theobservation $\xi_{k}$ is $\mathrm{Cov}(X_{k}|\xi_{k})$ with a mean of $x_{k}$. This covariance is estimated using the KF steps Equations (15) and (16). It is assumed that the estimation processes are independent between the time steps. Consequently, the necessary adjustments to the KKF filtering algorithm are made using a first-order Taylor-series expansion depending on the correlation rate of change during the Kriging step. The first-order approximation of the statistics measurements reveals a straightforward relationship with spatial errors stemming from the quadcopters’ position uncertainty, which is derived from the covariances in the MFC-KF estimation process. Therefore, it is also important to regard a field value as a random variable, $Y_{k}$, whose estimated value is an expectation conditioned upon the current measurements and the latest estimated values. That is,

(36) $${\hat{Y}}_{k}=E\left(Y_{k}|\xi_{k},Y_{k-1}\right).$$

In Section 2.6, the covariance $\Sigma_{k}^{+}$ of $y_{k}$ was essential for producing the KKF algorithm, but it did not exhibit a direct relationship to positional information. Since $\Sigma_{k}^{+}=\mathrm{Cov}\left(Y_{k}|\xi_{k}\right)$, this relationship can be unveiled by expanding the covariance as a function of the positional uncertainty and the spatial correlation of the field. This implies that

(37) $$\begin{array}{cc} \mathrm{Cov}(Y_{k}|\xi_{k})= & \;\mathrm{Cov}(Y_{k}|X_{k}=\xi_{k},Y_{k-1})\\ \; & +{\left(\frac{\partial E(Y_{k}|X_{k},Y_{k-1})}{\partial X_{k}}\right)}^{⊤}\mathrm{Cov}\left(X_{k}|\xi_{k}\right)\left(\frac{\partial E(Y_{k}|X_{k},Y_{k-1})}{\partial X_{k}}\right)\\ \; & +\mathrm{higher}\;\mathrm{order}\;\mathrm{terms}. \end{array}$$

**Figure 5:**
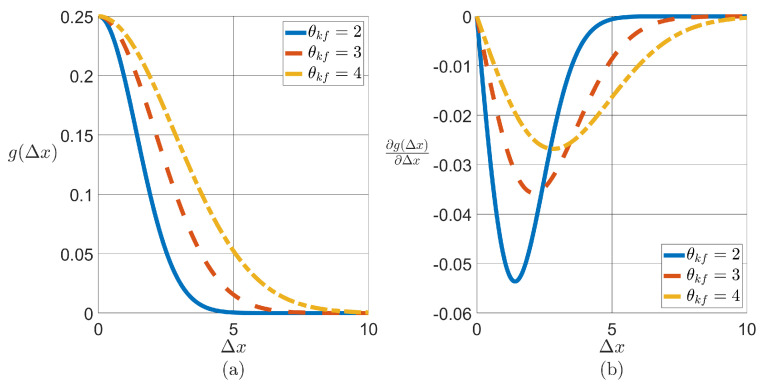
(**a**) Spatial correlation for three values of θ (**b**) and its corresponding derivatives.

The key observation in (37) is that the second term on the right-hand side connects the variance of the measurements with the covariance $\mathrm{Cov}(X_{k}|\xi_{k})$ obtained from the MFC-RGMKF approach. Moreover, $\frac{\partial E(Y_{t}|X_{t},Y_{t-1})}{\partial X_{t}}$ denotes the change in measurement value as a function of the spatial position of the acquisition platform. This change is given in terms of the derivative of the correlation function

(38) $${\mathrm{corr}}_{\theta_{kf}}\left(x_{i},x_{j}\right):=\sigma_{kf}^{2}e^{\left(-\frac{\left|\right|x_{i}-x_{j}{\left|\right|}_{2}^{2}}{\theta_{kf}^{2}}\right)},$$

where the parameter $\theta_{kf}$ controls the strength of the spatial correlation in the field of interest (see Figure 5 for three values of $\theta_{kf}$), $\sigma_{kf}$ is scale factor, and $\left|\right|\cdot {\left|\right|}_{2}$ denotes the standard Euclidan norm. For simplicity, assume that the correlation in the field is isotropic. Then, ${\mathrm{corr}}_{\theta_{kf}}\left(x_{i},x_{j}\right)=g\left(\Delta x\right)$ is only a function of the separation between $x_{i}$ and $x_{j}$, namely Δx, so its derivative is $\frac{\partial g(\Delta x)}{\partial \Delta x}$. Finally, the covariance of the measurements in the KKF algorithm given in (26) is then updated as $R_{k}=R_{y,k}+R_{y,x,k}$, where $R_{y,k}=\mathrm{Cov}\left(Y_{k}|X_{k}=\xi_{k},Y_{k-1}\right)$, $R_{y,x,k}={\left(\frac{\partial E(Y_{k}|X_{k},Y_{k-1})}{\partial X_{k}}\right)}^{⊤}\mathrm{Cov}\left(X_{k}|\xi_{k}\right)\left(\frac{\partial E(Y_{k}|X_{k},Y_{k-1})}{\partial X_{k}}\right)$, and $\mathrm{Cov}(X_{k}|\xi_{k})$ is the error covariance coming from the MFC-RGMKF algorithm, and $\frac{\partial E(Y_{k}|X_{k},Y_{k-1})}{\partial X_{k}}=\frac{\partial g(\Delta x)}{\partial \Delta x}$. The method to quantify the covariance from the quadcopter system running MFC-RGMKF for error quantification is referred to as MFC-KKF. Finally, it is important to mention that a KF has a time complexity of $O(n^{3})$ [49,50] and that the KKF is developed using a similar framework. Therefore, it is natural to expect an increase in computational demand as the number of grid points considered in the reconstruction of an information field becomes larger. One strategy to address this issue involves considering the sparsity in the field [51]. A similar conclusion can be reached when a swarm of quadcopters is used instead of a single quadcopter. Without diminishing the importance of these issues, addressing them is outside the scope of the results presented here and left for consideration in future work. The overall integration of the MFC-KKF procedure is summarized in Algorithm 3.
**Algorithm 3:** MFC-KKF
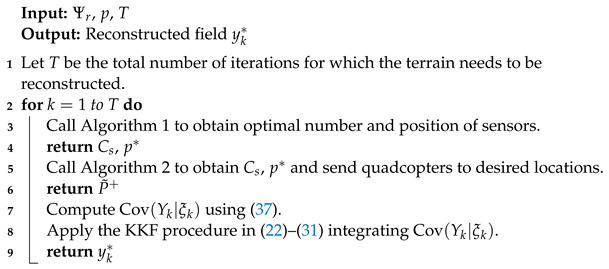


## 4. Numerical Simulations

This section delves into the outcomes of the methods outlined in Section 3. First, data reconstruction using the QR methodology is shown when compared to random sensor placement. Next, a simulation showing the quadcopter tracking with MFC-RGMKF is presented by comparing the advantage of the RGMKF against the use of the standard KF. Finally, a reconstruction using different uncertainty levels is shown with the purpose of illustrating how these affect the acquisition network.

### 4.1. Field Reconstruction

**Figure 6:**
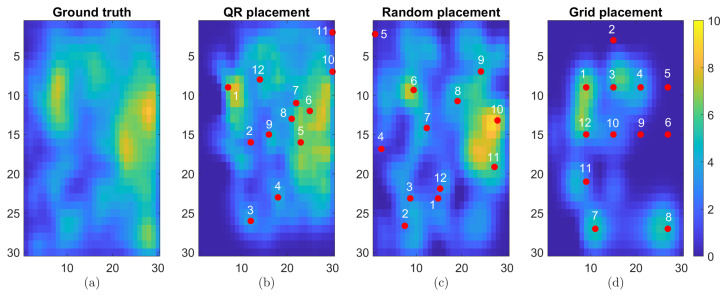
(**a**) Ground truth, (**b**) reconstruction using QR sensor placement, (**c**) reconstruction using random sensor placement, and (**d**) reconstruction using grid placement. The red dots indicate the locations where data points where obtained for the filed reconstruction procedure.

A synthetic field was generated on a 30×30 grid, comprising 60 time instances. An instance of the true synthetic field dataset is presented in Figure 6a with stationary component $s_{k}$ modeled by $s_{k}∼N\left(1_{N},\Sigma_{s}\right)$. The spatial distribution of the non-stationary component at the initial time and the Gaussian kernel function that models the state transition matrix are used as outlined in Section 2.6. The field evolves over space and time, represented as $u_{k}=v_{k}+s_{k}$.

**Figure 7:**
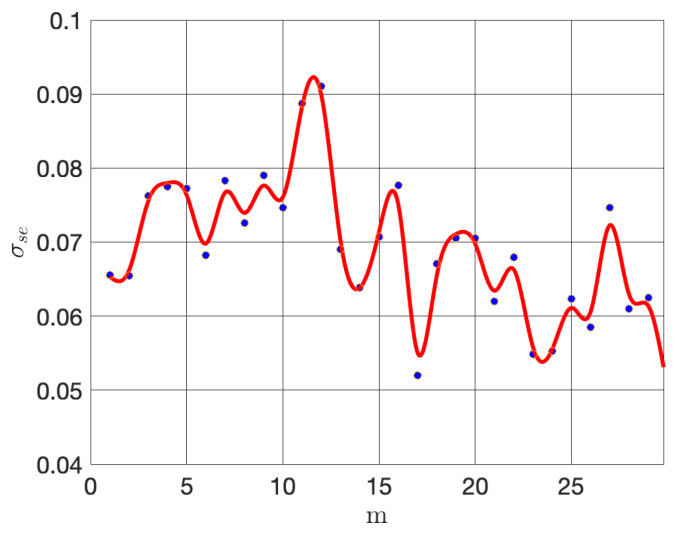
Minimum singular value for a maximum of m=30 sensors. The blue dots indicate singular values, and the red curve is the corresponding polynomial fitting showing their trend.

At each time instance of the reconstruction process, the optimal number of sensors has been determined as outlined in Section 3.1 for m=30. The trade-off between the number of sensors and reconstruction quality can be assessed using the minimum singular values of $M_{\gamma ,p}$. Figure 7 shows the plot of minimum singular values versus the number of sensors, which reveals that, although these values fluctuate, there is a region on the plot where the conditioning of $M_{\gamma ,p}$ is best (between p=11 and p=12). This pattern reflects the dynamic interaction between sensor placement and the characteristics of the measurement field. Recall also that considering more sensors increases the dimension of $M_{\gamma ,p}$, and thus an interplay between dimension and conditioning is exhibited in the experiment. In this case, the value $p^{*}=12$ is chosen, which balances the reconstruction cost against the sampling cost. Note that, in general, $p^{*}$ will depend on the particular phenomena producing the information obtained by the acquisition network.

The quality of the reconstruction process was also evaluated in Table 1 by using different numbers of sensors optimally placed utilizing the QR method. The quality was measured using the Root Mean Squared Error (RMSE), the Mean Absolute Error (MAE), and the Structural Similarity Index Measure (SSIM) between all grid values of the ground truth and the reconstruction. The expressions of these metrics are

$$\begin{array}{cc} \mathrm{MAE} & =\frac{\sum_{i=1}^{n}\left|y_{i}-x_{i}\right|}{n},\\ \mathrm{RMSE} & =\sqrt{\frac{\sum_{i=1}^{n}{\left(y_{i}-x_{i}\right)}^{2}}{n}},\\ \mathrm{SSIM} & =\frac{\left(2\mu_{x}\mu_{y}+C_{1}\right)\left(2\sigma_{xy}+C_{2}\right)}{\left(\mu_{x}^{2}+\mu_{y}^{2}+C_{1}\right)\left(\sigma_{x}^{2}+\sigma_{y}^{2}+C_{2}\right)}, \end{array}$$

where $C_{i}={\left(k_{i}L\right)}^{2}$ for i=1,2, and $L=2^{\#\mathrm{bits}\;\mathrm{per}\;\mathrm{pixel}}-1$.

Here $y_{i}$ denote the *i*-th value in the reconstructed grid or pixel, and $x_{i}$ the *i*-th value in the ground truth grid, *n* represents the total number of pixels in the image. The pixel sample means of the ground truth image *x* and the reconstructed image *y* are given by $\mu_{x}$ and $\mu_{y}$, respectively. The variances of the pixel values in *x* and *y* are $\sigma_{x}^{2}$ and $\sigma_{y}^{2}$, respectively, while $\sigma_{xy}$ represents the covariance between the pixel values in *x* and *y*. To stabilize the division when the denominator is close to zero, the constants $C_{1}$, $C_{2}$ are introduced, where $k_{1}=0.01$, $k_{2}=0.03$, and *L* represent the range of the pixel values. The latter metric measures the similarity between the original and reconstructed images by considering degradation as a perceived change in structural information [52] and ranges between −1 and 1, where 1 indicates perfect similarity, 0 indicates no similarity, and negative values indicate dissimilarity.

It can be observed that using fewer sensors than the optimal number results in poor reconstruction quality due to under-sampling. In this case, the collected data points are insufficient to accurately represent the field’s variations and details, leading to a loss of important information. For the optimal number of sensors in the last reconstructed field ($p^{*}=12$), a significant improvement is observed with errors that are nearly halved compared to lesser amount of sensor values. Observe that adding more sensors after $p^{*}$ does not significantly improve the reconstruction because it indicates the trade-off between sensor locations and the dimension of $M_{\gamma ,p}$. Also, in general, using a higher number of sensors might offer improvements, but such gains are not significant when compared to the configuration using the optimal number of sensors. For example, increasing the number of sensors from 10 to 12 resulted in a 1.42 improvement in RMSE. Interestingly, further increasing the number of sensors from 12 to 25 yielded only 0.9 improvement in RMSE.

The QR placement technique was also compared against random placement and uniform placement over the grid using $p^{*}=12$ sensors. MAE, RMSE, and SSIM were used again to assess the quality of the reconstruction. These metrics are provided in Table 2. From Figure 6b, it can be observed that the more relevant information is captured by the QR methodology, with few features missing. In contrast, the random placement cannot ensure the capture of such features despite having an apparently better coverage of the area as shown in Figure 6c. In Figure 6d, uniform placement over a grid shows that the reconstruction still cannot capture information as well as the QR method. All results in Figure 6 and Table 2 were obtained from the last time instance of the reconstruction.

### 4.2. Quadcopter Tracking

**Figure 8:**
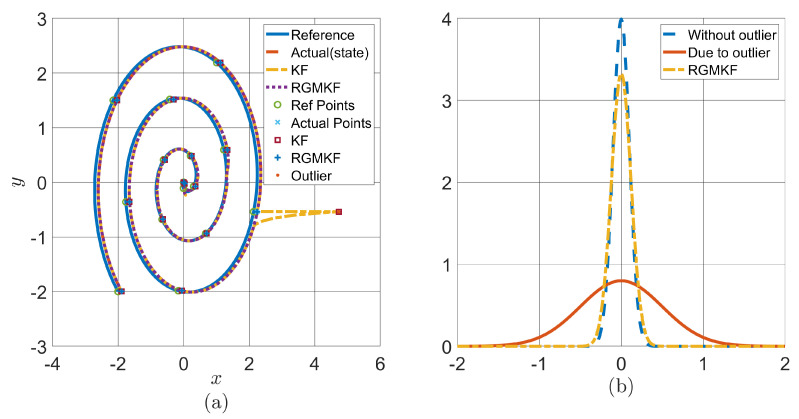
(**a**) Quadcopter tracking a spiral reference using MFC-KF and MFC-RGMKF. (**b**) Uncertainty quantification with and without an outlier using the MFC-KF and MFC-RGMKF.

To validate the proposed algorithms for quadcopter systems comprising the acquisition network, simulations were performed using the quadcopter parameters given in Table 3 and the MFC parameters given in Table 4. The purpose of this simulation is to highlight the importance of RGMKF, especially in the presence of outliers; its role in the rectification of such values; and the effect of uncertainty. A simulation for a quadcopter system tracking a spiral reference in a plane is shown in Figure 8. In this simulation, an outlier is introduced in the *x*-axis while the quadcopter tracks the desired spiral reference. Figure 8 compares the covariances of the KF and RGMKF. Figure 8a shows that the KF deviates significantly from the original state when the outlier occurs, whereas the RGMKF detects the outlier, rectifies it, and makes the quadcopter trajectory remain closer to the desired one. Figure 8b quantifies the positional uncertainty on the *x* coordinate for the case in which there is an outlier (blue), the case with an outlier using MFC-KF (orange), and the case with an outlier using MFC-RGMKF (yellow). The outlier adversely affects the KF’s covariance, and as such, the corresponding uncertainty has a wider spread, the RGMKF reduces the uncertainty spread, which gives a better representation of the positional uncertainty of the system.

### 4.3. MFC-KKF for the Reconstruction of a Synthetic Filed Information

**Figure 9:**
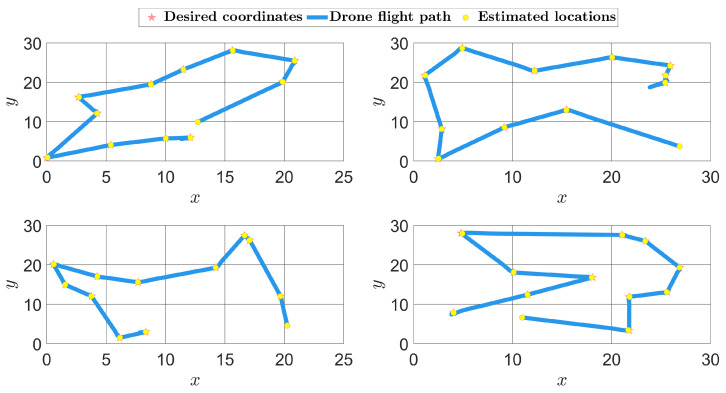
Four planned QR sensor placements’ flight paths overlaid with the simulated quadcopter path using MFC-KF with the RGMKF method.

**Figure 10:**
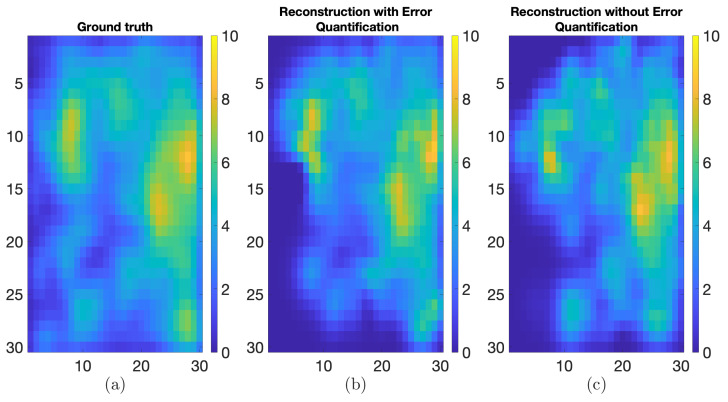
(**a**) Ground truth, (**b**) reconstructed data obtained using MFC-KKF, and (**c**) reconstructed data without using error quantification.

The synthetic field shown in Figure 6a was reconstructed using the MFC-KKF method with $p^{*}=12$ sensors at a position given by the QR methodology, where the quadcopter parameters used in the simulation of the acquisition network are given in Table 4. Four time instances of the quadcopter path are given in Figure 9. Furthermore, the reconstruction of the field with and without the positional error quantification, developed in Section 3.3, is given in Figure 10. The quality of the reconstruction is again presented in terms of the metrics used before and given in Table 5. Observe that all metrics improve after adding positional uncertainty to the reconstruction process.

**Figure 11:**
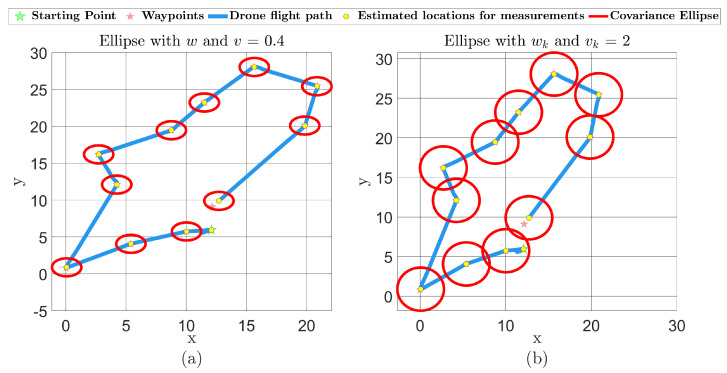
(**a**) shows a flight path with lower noise level, and (**b**) is the same path but with higher noise level.

Finally, it is natural that when the acquisition network performs with noisy environments, then the quadcopters position exhibit higher covariance, which is obtained using the MFC-RGMKF. Thus, another test was performed where the process and sensor noises in the quadcopter system were varied. The effect of changing the noise level can be seen in Figure 11b, where a significant change in the covariance ellipsoid is observed at every measurement location used for the reconstruction. The error metrics are given in Table 6. It can be noted that a higher noise level increases the reconstruction error. Additionally, the reconstruction metrics were compared against the case in which positional uncertainty is not considered. An improvement in all three criteria is observed when uncertainty integration is added for all three metrics considered.

## 5. Conclusions

This paper presents a comprehensive approach to spatio-temporal field reconstruction using a combination of optimal sensor placement, MFC, and the KKF. The key contributions of this study addressed challenges in field estimation under dynamic and uncertain conditions.

First, an optimal sensor-placement procedure was provided to determine the minimal number of sensors required for effective field reconstruction. This method, based on QR decomposition, ensures efficient sensor usage while maintaining high accuracy. Second, MFC was implemented on quadcopters to collect data from the selected optimal points, enhancing the system’s robustness against challenging external conditions. Finally, the covariance obtained from the MFC-RGMKF was integrated with the KKF to improve the reconstruction process, accounting for sensor variance and refining accuracy.

The reconstructions shown in Section 4 validated the effectiveness of these methodologies, demonstrating significant improvements in accuracy and efficiency. This study offers a robust framework for spatio-temporal field estimation, providing valuable insights for future research and applications in environmental monitoring, safety surveillance, and climate prediction.

## Figures and Tables

**Table 1:** Comparing the reconstruction of the field with a different number of sensors.

Number of Sensors	RMSE	MAE	SSIM
9	2.2527	1.3922	0.5021
10	2.8788	1.8647	0.4802
11	1.5635	1.0723	0.4529
12	1.4615	0.9806	0.4365
14	1.3158	0.7824	0.5702
16	1.0503	0.7103	0.5489
20	0.7675	0.5252	0.6451
25	0.5662	0.3439	0.7689
28	0.4087	0.2966	0.8115

**Table 2:** Metrics used for comparing the reconstruction between QR, random, and uniform placement.

Metric	QR Placement	Random Placement	Grid Placement
RMSE	1.4615	1.6722	3.0231
MAE	0.9806	1.1328	2.2484
SSIM	0.4365	0.3548	0.1968

**Table 3:** Parameters used in the quadcopter system and their values.

Parameter	Value
*g*	9.8
*m*	0.47
*l*	0.25
$K_{m}$	$3.0\times 10^{-5}$
$I_{xx}$	$3.8\times 10^{-3}$
$I_{yy}$	$3.8\times 10^{-3}$
$I_{zz}$	$7.7\times 10^{-3}$
*J*	$2.8\times 10^{-5}$
$k_{d}$[x,y,z]	$\left[5.6,\;5.6,\;6.4\right]\times 10^{-4}$
$k_{d}$[ϕ, θ, ψ]	$\left[5.6,\;5.6,\;6.4\right]\times 10^{-4}$
*w*	0.1
*v*	0.1

**Table 4:** Model-free control parameters and values.

Parameter	Value	Parameter	Value	Parameter	Value
$K_{px}$	3.4	$K_{p\phi }$	0.48	$\beta_{x}$	0.377
$K_{dx}$	3.5	$K_{d\phi }$	1.4	$\beta_{y}$	0.446
$K_{py}$	3.4	$K_{p\theta }$	0.48	$\beta_{z}$	2.075
$K_{dy}$	3.5	$K_{d\theta }$	1.4	$\beta_{\phi }$	65
$K_{pz}$	5.9	$K_{p\psi }$	1.87	$\beta_{\theta }$	65
$K_{dz}$	6.0	$K_{d\psi }$	3.0	$\beta_{\psi }$	91

**Table 5:** Error metrics used for comparing the reconstruction with and without quantification.

Metric	With Quantification	Without Quantification
RMSE	1.0896	1.1369
MAE	0.8017	0.8292
SSIM	0.5902	0.5754

**Table 6:** Error metrics used for comparing the reconstruction under several uncertainty levels.

Metric	w=v=0.4	w=v=2	No Position Uncertainty
RMSE	1.0416	1.0896	1.1369
MAE	0.6696	0.8017	0.8292
SSIM	0.5918	0.5902	0.5754

## Data Availability

Data are contained within the article.

## Nomenclature

*x*	Signal compressible representation.
Ψ	transformation basis.
ϱ	sparse vector indicating the active modes in Ψ, belonging to $\left(R^{n}\right)$.
*y*	Measurement vector.
*C*	Measurement matrix.
$\sigma_{se}$	Singular value.
*F*	Overall time-varying dynamics in MFC.
β	Tuning parameter in MFC.
(v)	Order of MFC systems.
$y^{\left(v\right)}$	*v*-th order output of the system.
$y_{d}^{\left(v\right)}$	*v*-th order desired reference output.
Σ	Covariance matrix.
*k*	Time Step.
$X_{k}$	True location of an observation at time *k*
$\xi_{k}$	Estimate of the position obtained through auxiliary data.
$corr_{\theta }\left(xi,xj\right)$	Spatial correlation function between positions (xi) and (xj).
$Cov(X_{k}|xi_{k})$	Variance of the true location $X_{k}$, conditional on the estimated position $\xi_{k}$.
$\sigma_{s}$	Scale factor for correlation.
(^)	Estimated states.
(˜)	Robustified states.
MFC	Model-Free Control.
KF	Kalman Filter.
KKF	Kriging Kalman Filter.
RGMKF	Robust Generalized Maximum Likelihood Kalman Filter.
POD	Proper Orthogonal Decomposition.
SVD	Singular Value Decomposition.
PS	Projection Statistics
